# Differences Between Patient and Surgeon Perspectives: A Long-Term Follow-Up of 180 Patients With Zygomaticomaxillary Complex Fractures Following Either Conservative or Surgical Treatment

**DOI:** 10.1177/19433875231208463

**Published:** 2023-10-27

**Authors:** Samin Rahbin, Ola Sunnergren, Ellen McBride, Hatef Darabi, Babak Alinasab

**Affiliations:** 1 Division of ENT Diseases, Department of Clinical Science, Intervention and Technology (CLINTEC), Karolinska Institutet, Stockholm, Sweden and Department of ENT Diseases, Karolinska University Hospital, Stockholm, Sweden; 2 Ear, Nose and Throat Clinic, Region Jönköping County, Jönköping, Sweden and Centre for Oral Health, Department of Odontology and Oral Health, School of Health and Welfare, Jönköping University, Jönköping, Sweden; 3 25545 The Public Health Agency of Sweden, Stockholm, Sweden

**Keywords:** malar asymmetry, facial asymmetry, patient satisfaction, zygomatic fracture, ZMC, conservative or surgical treatment

## Abstract

**Study Design:**

Retrospective with follow-up.

**Objective:**

This study described the long-term outcomes of patients who received either conservative or surgical treatment for zygomaticomaxillary complex (ZMC) fractures. It accounted for the perspectives of both patients and surgeons, and explored factors associated with patient satisfaction.

**Methods:**

Patients with unilateral ZMC fractures 2007–2018 were invited to follow-up clinical examinations and photographic documentation. Patient experiences were recorded using a questionnaire. A review panel assessed computed tomography (CT) scans and photographs. Patient and surgeon perspectives of detecting functional sequelae were assessed, and a correlation matrix was used to evaluate different perspectives of perceiving malar asymmetry.

**Results:**

The study sample consisted of 180 patients, of which conservative treatment was given to 43 patients and surgical treatment to 137 patients. Median follow-up time was 72.5 months after trauma. Overall satisfaction was 92.8%, with no significant difference between treatment groups. Patients and surgeons showed marked differences in detecting functional sequelae. Predicted malar asymmetry on CT scans did not correlate to findings on photographs or reports by patients.

**Conclusions:**

A predicted sunken cheek on CT imaging does not necessarily lead to long-term visible asymmetry of the malar region. Surgeons should acknowledge different perspectives when predicting and assessing long-term sequelae of ZMC fractures, and seek consensus on when to perform surgical reconstructions.

## Introduction

Zygomaticomaxillary complex (ZMC) fractures are among the most common types of maxillofacial fractures, primarily affecting males between the ages of 20 and 39.^1,2^ Patterns and types of dislocation can vary, leading to a range of functional and cosmetic sequelae.^3,4^ Treatment options are conservative (non-surgical) or surgical, such as closed reduction (CR) or open reduction with internal fixation (ORIF) using plates and screws.^5,6^

The decision to pursue surgical treatment is often taken during the patient-surgeon consultation and different perspectives are taken into consideration.^5,6^ It has been suggested that dislocated ZMC fractures can result in visible malar asymmetry, and that cases presenting with persistent trismus or diplopia require surgery.^6-8^ However, conservative treatment can be considered when risks of sequelae are mild and mainly cosmetic, such as minor asymmetry caused by a sunken or flattened cheek. In such cases, surgery is not always ideal, as it can lead to iatrogenic scar formations and further cosmetic deformities.^3-5,9^

There is a lack of studies describing conservatively treated ZMC fractures, their long-term outcome, and how sequelae are perceived by patients and surgeons.^10,11^ In the absence of a consensus on when and how to perform a surgical repair, treatment decisions are influenced by surgeon experience, knowledge, preference, and local traditions along with patient expectations and desires. Enhancing our understanding of these factors can help patients and surgeons make better treatment decisions for ZMC fractures.

The aim of this study was to describe the long-term outcomes of patients who received either conservative or surgical treatment for ZMC fractures, and to assess different perspectives of evaluating malar asymmetry, sensory disturbance, diplopia, and trismus. The perspectives of both patients and surgeons were taken into consideration, and factors associated with patient satisfaction were explored.

## Materials and Methods

### Study Design, Inclusion, and Exclusion

This retrospective study was based on medical records and was supplemented by a long-term follow-up with questionnaires and clinical examinations. It was conducted at the Karolinska University Hospital (KUH) in Stockholm, Sweden, in adherence to the Declaration of Helsinki, and was approved by the regional ethical review authority (Etikprövningsnämnden) in Stockholm (2018/302-31).

**Figure 1.**
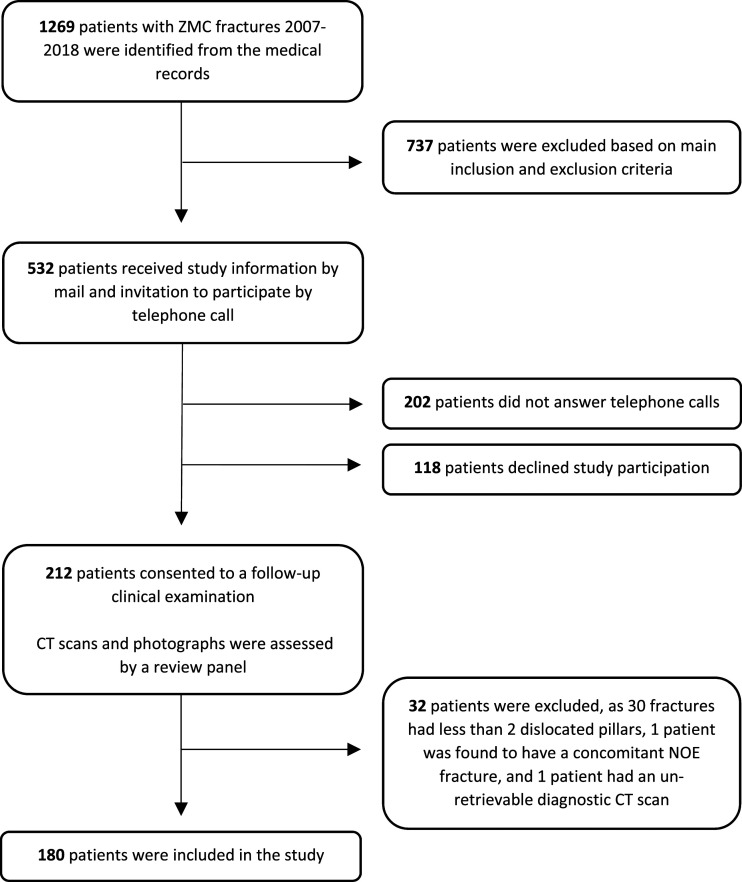
Flow chart illustrating the inclusion and exclusion process. Exclusion had to be performed at 3 stages: following application of main inclusion and exclusion criteria, before clinical examinations, and following review panel assessment. The study sample consisted of 180 patients. ZMC = zygomaticomaxillary complex; CT = computed tomography; NOE = Naso-orbito-ethmoidal.

Patients with ZMC fractures between 2007 and 2018 were identified using the diagnostic code S02.4 (fracture of malar and maxillary bones) of the 10^th^ edition of the International Classification of Diseases (Figure 1). The data included patients with ZMC fractures who received either conservative or surgical treatment under the care of the Department of Otorhinolaryngology or the Department of Reconstructive Plastic Surgery. Surgical treatment consisted of CR or ORIF, using the *MatrixMIDFACE*™ *Plating System* (DePuy Synthes), and was performed without adherence to a specific treatment protocol.

The following inclusion criteria were applied: patients with an isolated unilateral ZMC fracture, aged 18 years and above at the time of trauma, treated at KUH, and residing within one hour from the hospital at the time of follow-up. Patients were excluded from the study if their medical records contained an inaccurate address or telephone number, or if they had dislocated concurrent midfacial fractures (e.g., LeFort fractures), extensive midfacial soft tissue injuries, unretrievable CT scans, language difficulties, or significant co-morbidities preventing study participation (e.g., end-stage cancer or severe psychiatric disorder). Due to the coronavirus-19 (COVD-19) pandemic and the intention to conduct clinical examinations, some elderly patients above 70 years were also excluded, as this group was identified to be at risk of severe infection by the Public Health Agency of Sweden.

A surgical resident and a senior consultant classified the fractures in accordance with the classification of van Hout,^12^ which is largely based on Zingg from 1992.^4^ Both systems describe incomplete fractures (A), tetrapod fractures (B), and comminuted fractures (C). However, Zingg defines type A as fractures to 1 of the 3 zygomatic pillars (A1-A3), whereas van Hout defines it as fractures where at least 1 of the pillars remain intact. The choice of van Hout’s classification was made to include more patients at risk of acquiring post-traumatic malar asymmetry. As a result, patients with less than 2 dislocated zygomatic pillars were examined and assessed, but ultimately excluded from the data analysis.

Patients who met inclusion criteria were contacted via mail and informed about the purpose of the study and the prospect of participating in a follow-up clinical examination. Within one month, they were all telephoned up to three times. Those who responded and consented to participate were scheduled for a clinical examination and further data collection. During the visit, written informed consent was obtained from each participant.

### Follow-Up Clinical Examinations and Collection of Data

Follow-up clinical examinations were conducted consecutively between 2018 and 2020 by either a medical student (third author, EL) or a surgical resident (first author, SR). Their assessment of clinical findings was calibrated under the supervision of a senior consultant (senior author, BA). A description of collected data from questionnaires, clinical examinations, and review panel assessments is presented in Table 1. Throughout the study, malar asymmetry was defined as a sunken face/cheek.

**Table 1.** Outcomes Recorded From Questionnaires, Clinical Examinations, and Review Panel Assessments of CT Scans and Patient Photographs. CT = Computed Tomography; ION = Infraorbital Nerve; NA = Not Applicable.

	Questionnaire	Clinical assessment (standardized protocol)	Panel assessment (CT scans and photographs)
**Satisfaction with appearance**	Are you satisfied with how your injury appears today? Yes/No	NA	NA
**Malar asymmetry**	Is the injured side of your face/cheek visibly sunken? Yes/No	NA	Sunken face/cheek Yes/No^a,b^
**Sensory disturbance**	Has the injury caused a sensory disturbance in your face? Yes/No	*Cotton swab test of the ION* decreased sensation at 2 out of 3 locations (eyelid, nose, lip) Yes/No	NA
**Diplopia**	Has the injury caused double vision? Yes/No	*Eye motility examination* Diplopia when following the examiner’s finger in an “H” pattern Yes/No	NA
**Trismus**	Has the injury caused difficulties when mouth opening? Yes/No	Self-reported struggle to open mouth + inter-incisal distance Yes/No	NA
**Enophthalmos**	NA	NA	Visual assessment Yes/No^b^

^a^Assessed on diagnostic and post-operative CT scans.

^b^Assessed on patient photographs.

To begin with, the participants completed a self-created questionnaire about their satisfaction with facial appearance and their opinion on whether the injury had caused malar asymmetry, sensory disturbance, diplopia, or trismus (mouth-opening difficulties).

A clinical assessment followed, noting the presence of trismus, diplopia, and sensory disturbance (decreased sensation) corresponding to the infraorbital nerve (ION). Trismus was defined as a self-reported struggle to open the mouth during the examination. In such cases, inter-incisal distance was measured. Diplopia was assessed by examining eye motility, with the patient gazing on the examiner’s finger and following its movements in an “H” pattern. The ION was tested by gently brushing a cotton swab over the lower eyelid, wing of the nose, and upper lip while comparing the injured side of the face to the uninjured side. A decreased sensation was documented when patients reported hypoesthesia or anesthesia on the injured side, on at least 2 of the 3 locations.

A Digital Single-Lens Reflex (DSLR) camera with an 18–55 mm lens was used to photograph all study participants. To adequately capture facial disfigurements, images were taken with the head positioned in 5 angles: frontal, basal, cephalic, oblique, and lateral.

The following patient data were finally extracted from the medical records: patient ages at the time of trauma and follow-up, gender, date of first visit, fracture cause and classification, and type of treatment (conservative or surgical). All diagnostic CT scans of the facial skeleton taken during the initial encounter, and those taken post-operatively, were rendered into 3-dimensional (3D) models and saved in the same 5 angles as the patient photographs.

### Review Panel Assessment

In an effort to reduce subjective bias, a review panel consisting of 3 senior surgeons, actively treating maxillofacial fractures at KUH, was formed to review CT scans and photographs. Panel outcome was decided by majority vote.

The CT scans and patient photographs were de-identified and sorted into 3 different files using PowerPoint (Microsoft Office, version 2212). Each file contained slides with either diagnostic scans of all patients, available post-operative scans of patients with surgical treatment, or photographs of all patients. They were presented during 1 of 3 separate panel sessions. In between each session, the patient order of display was altered. Panel members were only provided information on laterality of a ZMC fracture.

During the first 2 panel sessions, all diagnostic and available post-operative CT scans were shown. The panel was asked to predict whether the appearance of the displayed zygomatic complex would result in malar asymmetry, assuming that no (further) surgery was to be performed. During the third session, when patient photographs were shown, the panel was asked to indicate whether malar asymmetry or enophthalmos was present on the injured side of the face.

### Statistical Analysis

Descriptive data was presented as total number (n), percentage of group (%), median, and interquartile range (IQR). Comparisons between 2 groups were analyzed using the non-parametrical Mann-Whitney U test. Dichotomous data was analyzed using Pearson’s chi-square test, or Fisher’s exact test when statistical assumptions were violated. Additionally, a Pearson’s correlation matrix was created to analyze malar asymmetry. Odds ratios (OR) and confidence intervals (CI) were calculated using uni- and multivariate logistic regression. Statistical significance was set at the level of *P* < .05. All statistical analysis was performed using IBM SPSS Statistics for Windows version 28 (IBM Corp., Armonk, NY, USA).

## Results

Following application of main inclusion and exclusion criteria, 532 patients were contacted to be invited to the study (Figure 1). A total of 320 patients (60.1%) were excluded from the study due to non-response (n = 202, 37.9%) or refusal to participate (n = 118, 22.2%). An additional 32 patients (6.0%) were excluded from data analysis after the fractures were classified. The remaining 180 patients (33.9%) were included in the study.

Of the 532 patients invited to participate, no significant differences were noted between included and excluded patients with regards to median ages at trauma (44.0 vs 39.5 years, *P* = .055), follow-up (50.0 vs 46.0 years, *P* = .235), or gender distribution (80.6% vs 80.2% males, *P* = .916, data not shown).

Table 2 (sorted by treatment type) and Table 3 (sorted by fracture classification) present the distribution of findings. Patients were examined after a median of 72.5 months following trauma. The majority of study participants were male (n = 145, 80.6%) and were classified with type B fractures (n = 92, 51.1%). Falls (n = 62, 34.4%; 62.9% male) were the most common cause of injury, followed by inter-personal violence (n = 49, 27.2%; 100.0% male), and bicycle accidents (n = 30, 16.7%; 76.7% male, data not shown).

**Table 2.** Outcomes Sorted by Treatment type. Findings are Expressed as Total Numbers (% of Group), Except for Ages at Trauma and Follow-Up (Median Years, IQR), and Length of Follow (Median Months, IQR). CT = Computed Tomography; IQR = Interquartile Range; NA = Not Applicable.

Variable of Interest	Conservative treatment (n = 43)	Surgical treatment (n = 137)	Total study sample (n = 180)
Male gender	31 (72.1)	114 (83.2)	145 (80.6)
Age at trauma	51.0 (37.5-62.0)	42.0 (28.0-52.0)	44.0 (28.0-54.5)
Age at follow-up	57.0 (44.5-66.0)	48.0 (34.0-58.0)	50.0 (34.0-59.5)
Length of follow-up	61.0 (28.5-86.0)	76.0 (38.0-104.0)	72.5 (36.0-96.5)
*Fracture classification*			
A	24 (55.8)	32 (23.4)	56 (31.1)
B	15 (34.9)	77 (56.2)	92 (51.1)
C	4 (9.3)	28 (20.4)	32 (17.8)
Questionnaire			
Satisfaction	37 (86.0)	130 (94.9)	167 (92.8)
Malar asymmetry	6 (14.0)	15 (10.9)	21 (11.7)
Sensory disturbance	19 (44.2)	85 (62.0)	104 (57.8)
Trismus	8 (18.6)	10 (7.3)	18 (10.0)
Diplopia	5 (11.6)	4 (2.9)	9 (5.0)
Clinical examination			
Sensory disturbance	16 (37.2)	74 (54.0)	90 (50.0)
Trismus	3 (7.0)	4 (2.9)	7 (3.9)
Diplopia	1 (2.3)	4 (2.9)	5 (2.8)
CT scan assessment			
Malar asymmetry (diagnostic scan)	19 (44.2)	114 (83.2)	133 (73.9)
Malar asymmetry (post-operative scan)	NA	24 (17.5)	24 (13.3)
Post-operative scan not performed	NA	31 (22.6)	31 (17.2)
Photograph assessment			
Malar asymmetry	15 (34.9)	37 (27.0)	52 (28.9)
Enophthalmos	3 (7.0)	16 (11.7)	19 (10.6)

**Table 3.** Outcomes Sorted by Fracture Classification. Findings are Expressed as Total Numbers (% of Group). CT = Computed Tomography.

Variable of Interest	Fracture type A (n = 56)	Fracture type B (n = 92)	Fracture type C (n = 32)
Questionnaire			
Satisfaction	53 (94.6)	86 (93.5)	28 (87.5)
Malar asymmetry	7 (12.5)	9 (9.8)	5 (15.6)
Sensory disturbance	30 (53.6)	54 (58.7)	20 (62.5)
Trismus	6 (10.7)	7 (7.6)	5 (15.6)
Diplopia	3 (5.4)	2 (2.2)	4 (12.5)
Clinical examination			
Sensory disturbance	24 (42.9)	52 (56.5)	14 (43.8)
Trismus	1 (1.8)	3 (3.3)	3 (9.4)
Diplopia	4 (7.1)	0 (.0)	1 (3.1)
CT scan assessment			
Malar asymmetry (diagnostic)	32 (57.1)	71 (77.2)	30 (93.8)
Malar asymmetry (post-operative)	3 (5.4)	15 (16.3)	6 (18.8)
Post-operative scan not performed	10 (17.9)	12 (13.0)	9 (28.1)
Photograph assessment			
Malar asymmetry	12 (21.4)	29 (31.5)	11 (34.4)
Enophthalmos	1 (1.8)	11 (12.0)	7 (21.9)

Conservative treatment was given to 43 patients (23.9%) and surgical treatment to 137 patients (76.1%). Patients with conservative treatment sustained higher rates of type A fractures (55.8% vs 23,4%, *P* < .001), and were older at the time of trauma (51.0 vs 42.0 years, *P* = .007) and follow-up (57.0 vs 48.0 years, *P* = .004). Patients with surgical treatment had more type B fractures (56.2% vs 34.9%, *P* = .015) and type C fractures (20.4% vs 9.3%, *P* = .096), and were more often male (83.2% vs 72.1%, *P* = .108).

### Sensory Disturbance, Trismus, and Diplopia

Patients with type C fractures exhibited the highest rates of patient-reported trismus (15.6%), patient-reported diplopia (12.5%) and trismus detected at clinical examinations (9.4%). 

Overall, sensory disturbance was the most common sequelae, reported by 104 patients in questionnaires (57.8%) and 90 patients at clinical examinations (50.0%). Clinical sensory disturbance was more often found in patients with type B fractures (n = 52, 56.5%) and after surgical treatment (n = 74, 54.0%). A total of 79 cases of patient-reported sensory disturbance were verified during examinations, corresponding to 76.0% of patient-reported cases and 87.8% of clinical cases. Patient-reported and clinically detected sensory disturbances exhibited a significant, moderate correlation (r = 0.607, *p* < 0.001).^1^

**Table 4.** Outcomes of Sensory Disturbance, Trismus, and Diplopia From Patient Questionnaires and Clinical Examinations. Findings Presented as Total Numbers (n) and Percentage (%) of Total Study Sample (n = 180). **A.** Sensory Disturbance (SD) **B.** Trismus (T) **C.** Diplopia (D).

		Clinical examination	
		*SD+*	*SD-*
Questionnaire	*SD+*	79 (43.9)	25 (13.9)
	*SD-*	11 (6.1)	65 (36.1)

		Clinical examination	
		*T+*	*T-*
Questionnaire	*T+*	7 (3.9)	11 (6.1)
	*T-*	0 (.0)	162 (90.0)

		Clinical examination	
		*D+*	*D-*
Questionnaire	*D+*	1 (.6)	8 (4.4)
	*D-*	4 (2.2)	167 (92.8)

Out of the 18 patients with patient-reported trismus, 7 patients confirmed trismus during examinations, and 3 of them measured an inter-incisal distance <35 mm (Table 4B). Of the 9 patients who reported diplopia, 1 patient could be confirmed on exam (Table 4C).

### Malar Asymmetry

Surgical treatment was significantly associated with a retrospective prediction of malar asymmetry on diagnostic CT scans (83.0% vs 44.2%, *P* < .001). Post-operative scans were not performed on 31 patients (22.6%). In relation to the remaining 106 surgically treated patients, a significant association was only observed with the length of follow-up (108.0 vs 61.5 months, *P* < .001, data now shown).

The sample used to evaluate for malar asymmetry consisted of patients with available CT scans, assumed to reflect the current morphology of the facial skeleton. A total of 149 patients, either conservatively treated with a diagnostic scan or surgically treated with a post-operative scan, were included in this part of the analysis. Within this group, 43 patients (28.9%) were predicted to suffer from malar asymmetry on CT scans. Among these patients, the panel detected 16 cases (37.2%) of malar asymmetry on photographs and 7 cases (16.3%) on patient questionnaires. Panel-evaluated scans, photographs, and patient questionnaires all found the highest rates of malar asymmetry among patients with conservative treatment (n = 19, 44,2%; n = 15, 34.9%; and n = 6, 14.0%, respectively).

A correlation matrix of the three perspectives to evaluate malar asymmetry is presented in Table 5. A significant, low correlation was found between patient-reported malar asymmetry and the photographic assessments of malar asymmetry by the review panel (r = .303, *P* < .001). However, neither patient reports nor photographic assessments of malar asymmetry were significantly correlated to predictions of malar asymmetry on diagnostic and post-operative CT scans (r = .067, *P* = .414 and r = .097, r = .238, respectively).

**Table 5.** Correlation Matrix of the Three Perspectives Used to Evaluate Malar Asymmetry. As 31 Surgically Treated Patients did Not Perform a Post-operative CT Scan, Only 149 Patients Could be Used for the Analysis (43 Conservatively Treated Patients With Diagnostic Scans and 106 Surgically Treated Patients With Post-operative Scans). Significance Set at 5% (*P* < .05). CT = Computed Tomography; PCC = Pearson Correlation Coefficient; n = Total Numbers.

		Predictions on CT scans	Patient-reported outcomes	Photo-evaluated outcomes
**Predictions on CT scans**	*PCC*		.067	.097
	*P-value*		.414	.238
	*n*		149	149
**Patient-reported outcomes**	*PCC*	.067		.303
	*P-value*	.414		**< .001**
	*n*	149		180
**Photo-evaluated outcomes**	*PCC*	.097	.303	
	*P-value*	.238	**< .001**	
	*n*	149	180	

### Patient Satisfaction

Most patients (n = 167, 92.8%) reported satisfaction with their appearance at follow-up, with higher rates among patients with surgical treatment (94.9% vs 86.0%, *P* = .084). No significant associations were found between patient satisfaction and ages at trauma (*P* = .505), follow-up (*P* = .412), or length of follow-up (*P* = .406).

In a univariate analysis (Table 6), patient satisfaction was found to be associated with 4 variables: patient-reported malar asymmetry (*P* < .001), patient-reported trismus (*P* < .001), clinical trismus (*P* = .047), and malar asymmetry detected through photographic assessments (*P* = .003). These associations remained significant after controlling for age and gender. However, when all 4 variables were entered into an adjusted multivariate logistic regression, only patient-reported malar asymmetry (OR .084, 95% CI .017–.417, *P* = .002) and patient-reported trismus (OR .042, 95% CI .005–.329, *P* = .003) showed significant associations. All of the aforementioned 4 variables were negatively associated with patient satisfaction.

**Table 6.** Significant Associations and ORs Found Between all Variables and Patient Satisfaction, Sorted by Type of Statistical test. Significance Set at 5% (*P* < .05). OR = Odds Ratio; CI = Confidence Interval.

Variable of Interest	Univariate logistic regression		Adjusted univariate logistic regression^a^		Adjusted multivariate logistic regression^b^	
	*OR (95%CI)*	*P-value*	*OR (95%CI)*	*P-value*	*OR (95%CI)*	*P-value*
Patient-reported malar asymmetry	.078 (.023-.266)	**< .001**	.065 (.018-.238)	**< .001**	.084 (.017-.417)	**.002**
Patient-reported trismus	.090 (.026-.312)	**<.001**	.060 (.015-.244)	**< .001**	.042 (.005-.329)	**.003**
Clinical trismus	.170 (.030-.977)	**.047**	.159 (.026-.964)	**.045**	2.099 (.138-31.813)	.593
Photographic assessments of malar asymmetry	.154 (.045-.526)	**.003**	.160 (.046-.553)	**.004**	.338 (.074-1.535)	.160

^a^Model has been adjusted for age at trauma, age at follow-up, and gender.

^b^Model includes all 4 significantly associated variables and has been adjusted for age at trauma, age at follow-up, and gender.

## Discussion

In this study, we described the outcomes of 180 patients with ZMC fractures following either conservative or surgical treatment. Marked differences were observed in the way patients and surgeons perceived sequelae; clinical examinations did not verify all cases of patient-reported functional sequelae, and patients did not perceive malar asymmetry to the same extent as surgeons. Furthermore, our findings showed that a predicted sunken cheek on CT imaging did not always result in long-term, visible asymmetry of the malar region.

### Sequelae and Long-Term Patient Satisfaction

Functional sequelae resulting from ZMC fractures include sensory disturbance, trismus, and diplopia, with the most common cosmetic sequelae being malar asymmetry and enophthalmos.^6^ As fracture patterns become more complex, sequelae tend to increase.^12^ In our study, type C fractures were more likely to cause long-term malar asymmetry, trismus, and enophthalmos, findings that are consistent with the literature. However, only 14 patients (43.8%) with type C fractures suffered clinical sensory disturbance, a lower rate than found in patients with type B fractures (n = 52, 56.5%). Studies have previously reported a difference in the rate of sensory disturbance between type B and C fractures and hypothesized a possible mechanism of absorption of the force of impact, resulting in comminution of the zygomatic complex and sparing—or possibly decompression—of the ION.^4,12^

Previous studies have reported higher rates of sensory disturbance (12–41%) and enophthalmos (0–14%) in patients with surgical treatment, and more malar asymmetry in patients with conservative treatment (3–24%).^8,13-15^ Few patients with diplopia and trismus have been described. Our findings are consistent with these studies, although unexpectedly many cases of malar asymmetry were observed, particularly among patients with conservative treatment (patient-reported n = 6, 14.0%; predicted on CT scans n = 19, 44.2%; photographic assessments n = 15, 34.9%). These results emphasize the importance of understanding when surgical treatment should be considered. However, caution should be exercised when comparing findings across studies, due to variations in ZMC fracture patterns and methods of evaluating sequelae.^3,16^ Furthermore, it should be noted that our study assumed fracture stability over time, as repeat CT scans were not obtained at follow-up. The effect of masticatory muscles on the stability of ZMC fractures has been debated in previous research.^3,17,18^

Prior studies have reported that patients with ZMC fractures typically experience poor health related quality of life (HRQoL) before and immediately after surgical treatment, followed by gradual improvement, reaching (or exceeding) the levels of the general population.^19-21^ Contrary to the broader perception of overall life quality, our study specifically focused on satisfaction of facial appearance reported by patients who underwent different types of treatment. Nevertheless, the high rate of satisfaction reported for the entire study sample (n = 167, 92.8%) can be indicative of a high HRQoL. These results are also consistent with the findings of Kaukola et al,^19^ where most patients (98.0%) reported post-operative facial appearance as moderate or good, and Kurita et al,^22^ who noted low post-operative patient annoyance with deformity (12.0%).

Satisfaction rates were slightly lower among patients receiving conservative treatment, although the difference was not significant (86.0% vs 94.9%, *P* = .084). This could be partly explained by the presence of more sequelae, especially malar asymmetry. However, selection bias can also be attributed. Similar to the findings of Kurita et al^22^ and Folkestad et al,^23^ our study primarily found that patient-reported sequelae were the factors negatively associated with patient satisfaction.

### Patient Characteristics

The median age at the time of trauma trauma (44.0) and gender distribution (80.6% males) found in this study were consistent with previous reports from Northern Europe.^19,23,24^ When further comparing the 2 treatment groups, distribution of fracture types and patient characteristics were similar with findings of earlier studies, suggesting that surgically treated patients were more likely to have displaced and complex fractures, be male, and belong to a younger age group.^8,11,25,26^ Inter-personal violence was only the second most common cause of injury (27.2%) and none of the patients were female. A similar pattern was also observed in a retrospective study by Salentjin et al,^25^ where only 5 of 58 patients (8.6%) with ZMC fractures resulting from inter-personal violence were female. This may indicate that females who experience assaults might be underrepresented in retrospective studies.

The length of follow-up was the only variable significantly associated with surgically treated patients who did not undergo a post-operative CT scan (*P* < .001). This reflects a gradual shift in practice of routinely performing post-operative scans. A significant association was also found between surgery and retrospective predictions of malar asymmetry on diagnostic CT scans (*P* < .001). Although reliable data on pre-operative sequelae was not available, this finding suggests that surgery was mainly performed for cosmetic reasons, as trismus and diplopia are considered surgical indications only when found persistent.^6,8,9^

### Patient and Surgeon Perspectives

Many ZMC fractures—particularly those without severe dislocation of the orbital floor—concern the visible symmetry of the malar region, and the challenge is to determine whether surgery is needed to avoid a sunken, flattened or widened cheek. Several factors can influence the decision, for example, the age, soft-tissue composition, and preference of a patient, along with the natural asymmetry of the human face, and differences in perspectives of evaluating facial asymmetry.

Most studies that describe post-traumatic malar asymmetry have primarily focused on post-operative CT scans, fracture reduction, and residual dislocation. Very few have considered the presence of skeletal asymmetry in conjunction with clinical exam findings or patients’ self-perception of facial appearance. In this regard, our study considers the 3 most common perspectives when evaluating malar asymmetry: the patient over the long-term, the clinical assessment of the surgeon, and predictions based on CT scan reviews.

Some discrepancies have previously been observed between dislocation detected on CT scans and the clinical presentation of malar asymmetry. For example, Lehtinen et al^27^ found that only 55 out of 95 patients with dislocated ZMC fractures on CT scans exhibited clinical asymmetry. Starch-Jensen et al^15^ noted clinical asymmetry in 13% of patients with satisfying facial contours on CT scans. Ellis and Kittidumkerng^3^ described that 2 out of 5 surgically treated patients with residual dislocation showed no visible asymmetry on patient photographs, suggesting that a certain degree of dislocation on CT scans may prove to be clinically insignificant, partly due to soft tissue masking.

In our study, less than half of the patients with predicted malar asymmetry on CT scans were confirmed on photographic assessments (n = 16/43, 37.2%). No significant correlations were found between such predictions and patient reports or photographic assessments, respectively. These findings not only reaffirm, but further stress that dislocation and predicted malar asymmetry on CT scans do not necessarily result in subjective or clinical asymmetry. Furthermore, despite a weak correlation between patient reports and photographic assessment of malar asymmetry (r = .303, *P* < .001), our findings indicate that malar asymmetry is more frequently observed by surgeons than reported by the patients themselves.

Patient-reported sensory disturbance showed a moderate correlation with findings on clinical examinations (r = .607, *P* < .001). There are several reasons why a stronger correlation could not be established. It was observed that patients occasionally reported sensory disturbances unrelated to the ION, such as plate discomfort and cutaneous nerve damage. Also, while the use of a cotton swab was a fast and simple method to clinically assess hypoesthesia and anesthesia, it may not fully capture the extent of ION damage. A more comprehensive and resourceful method would be needed to detect asymmetrical sensations across the entire area supplied by the ION, including the vestibule, and reveal additional signs of nerve damage, like thermal discomfort, hyperalgesia, and neuropathic pain.

Differences were also found in the way patients and surgeons reported other functional sequelae. Out of the 18 patients who reported mouth-opening difficulties, only 3 patients were found to have trismus with an inter-incisal distance of <35 mm. Several patients complained of discomfort from the temporomandibular joint area. Moreover, only 1 out of 9 cases of patient-reported diplopia could be confirmed with an eye motility exam.

In summary, we found that surgeons tend to overstate malar asymmetry, whereas patients more frequently report functional sequelae such as sensory disturbance, trismus, and diplopia. This has clinical implications on the patient–surgeon consultation, the setting where a decision is taken on whether to perform surgery. It is crucial to bear in mind that patients may not perceive malar asymmetry as surgeons do, a contrast especially relevant to the many cases of ZMC fractures with primarily cosmetic sequelae. Furthermore, clinically verified trismus and diplopia are rarely found as long-term sequelae, and certain causes of sensory disturbances can be difficult to predict and may not be avoidable.

In our experience, good patient information—before a final decision on treatment is made—is critical for patient satisfaction.

### Strengths and Limitations

A strength of our study is the large sample of 180 patients, consisting of patients with both surgical and conservative treatments for ZMC fractures. The evaluations were conducted a median of 6 years following trauma, accounting for the perspectives of both patients and surgeons. However, there are also several limitations to consider. First, the study’s retrospective design and dropout rate of 60.1% should be acknowledged, although no systematic biases were identified. The use of a non-validated questionnaire to assess patient satisfaction and perception of sequelae is another limitation. Furthermore, it can be noted that the review panel’s assessment of cosmetic sequelae was based on photographs, rather than panel members being present during the clinical examinations or basing their outcomes on more sophisticated methods to capture facial features.

## Conclusion

Differences have been observed in the ways patients and surgeons perceive sequelae following ZMC fractures. Predicted malar asymmetry on CT scans do not correlate with self-evaluations of patients or findings from clinical examinations. Most patients with ZMC fractures are satisfied with facial appearance after a median of 6 years, irrespective of treatment type. Surgeons should acknowledge different perspectives when predicting and assessing long-term sequelae and seek consensus on when to perform surgical reconstructions.

## Notes

1 This sentence has been updated to include 87.8% of clinical cases since original publication of this article.

2 Acknowledgments section has been updated since original publication of this article.
